# Characterization of Musty Odor-Producing Actinomycetes from Tropics and Effects of Temperature on the Production of Musty Odor Compounds

**DOI:** 10.1264/jsme2.ME17109

**Published:** 2017-10-31

**Authors:** Nurul Syahirah Shamsol Anuar, Aeyshah Abang Kassim, Motoo Utsumi, Koji Iwamoto, Masafumi Goto, Kazuya Shimizu, Nor’azizi Othman, Zuriati Zakaria, Norio Sugiura, Hirofumi Hara

**Affiliations:** 1 Department of Environmental Engineering and Green Technology, Malaysia-Japan International Institute of Technology (MJIIT), Universiti Teknologi Malaysia (UTM) Kuala Lumpur Malaysia; 2 Department of Chemical Process Engineering, Malaysia-Japan International Institute of Technology (MJIIT), Universiti Teknologi Malaysia (UTM) Kuala Lumpur Malaysia; 3 Department of Mechanical Precision Engineering, Malaysia-Japan International Institute of Technology (MJIIT), Universiti Teknologi Malaysia (UTM) Kuala Lumpur Malaysia; 4 Graduate School of Life and Environmental Science, University of Tsukuba Ibaraki Japan

**Keywords:** actinomycetes, musty odor compounds, tropical country

## Abstract

Geosmin and 2-methylisoborneol (MIB) outbreaks in tropical water bodies, such as Southeast Asia, by actinomycetes have not yet been elucidated in detail. Six *Streptomyces* isolates from lowland environments in Malaysia were selected and evaluated for their odor production under different temperatures. The gene responsible for the production of geosmin, *geoA*, was detected in all isolates, while only two isolates harbored *tpc*, which is responsible for 2-MIB production. This result suggested that geosmin and 2-MIB synthesis pathway genes already existed in the environment in the Tropics of Southeast Asia. Furthermore, our isolates produced musty odor compounds at 30°C, and differences were observed in musty odor production between various temperatures. This result indicated the potential for odor episodes in water bodies of the tropical countries of Southeast Asia throughout the year due to the mean annual ambient temperature of 27°C in the lowlands.

The presence of taste and odor compounds, particularly geosmin and 2-methylisoborneol (2-MIB), has been associated with odor episodes in freshwater worldwide. Their unpleasant taste and odor reduces public trust in drinking water and results in additional costs for water utilities as well as the loss of market demands in the aquaculture industry. These terpenoid compounds are produced as a secondary metabolite by microorganisms including actinomycetes (8, 9), cyanobacteria (12, 21, 23, 25, 27), myxobacteria (6, 34), fungi (16, 20), and some plants (red beets) (18). They are semi-volatile tertiary alcohols with low odor threshold concentrations of 4 ng L^−1^ and 9 ng L^−1^ for geosmin and 2-MIB, respectively (39). Due to their stable characteristics, conventional treatments such as coagulation, filtration, and sedimentation are not sufficient for their removal. A clearer understanding of the mechanisms underlying odor production is needed as the first step in developing a cost-effective removal strategy for these odorous compounds (33).

Actinomycetes, particularly *Streptomyces*, are the main producers of geosmin and 2-MIB in aquatic and terrestrial environments. Gerber and Lechevalier (8) were the first to identify geosmin from *Streptomyces griseus* LP-16 and 2-MIB was subsequently identified. Some *Streptomyces* spp. produce both compounds, whereas others only produce one (28). There are two common pathways for the biosynthesis of geosmin and 2-MIB: the 2-methylerythritol-4-phosphate (MEP) pathway and mevalonate (MVA) pathway. The MEP pathway is considered to be the major biosynthetic pathway for the production of geosmin and 2-MIB by *Streptomyces*. This pathway produces isopentyl diphosphate (IPP), which acts as an intermediate in the biosynthesis of isoprenoids. Geosmin is produced by the cyclization of farnesyl diphosphate (FPP) to germacradienol, and geosmin is then produced from germacradienol by geosmin synthase encoded by *geoA* (10). The compound 2-MIB is synthesized by the methylation of geranyl diphosphate (GPP) to produce 2-methyl GPP and the cyclization of 2-methyl GPP to produce 2-MIB, which is catalyzed by 2-MIB synthase (14).

Odor production by actinomycetes may be influenced by several environmental factors such as oxygen concentration, light intensity, carbon sources, pH, micronutrients, and temperature (7, 26, 29). It is important to note that the production of geosmin and 2-MIB in laboratory cultures may vary in response to numerous factors, and different *Streptomyces* spp. produce odorous compounds at different rates. Musty odor compounds have mostly been investigated in temperate and subtropical regions such as Europe, Japan, China, and North America, in which warmer temperatures are regarded as the key driver for the growth of *Streptomyces* and odor production during summer. The genes responsible for the production of geosmin and 2-MIB have also been detected in these regions from environmental samples. Therefore, temperature is considered to be an important environmental factor that affects the metabolic activity of *Streptomyces* (1) because of the temperature dependency of microbial growth.

To the best of our knowledge, the environmental factors affecting geosmin and 2-MIB production by actinomycetes in Southeast Asia have not yet been examined. In Malaysia, few studies have been conducted on musty odor compounds. Most studies investigated the detection of odorous compounds in freshwater fish (5, 24), but provided no information on potential actinomycetes producers or key environmental parameters affecting the production of geosmin and 2-MIB. Since Malaysia has a moderate tropical climate with a mean annual ambient temperature of 27°C and diurnal fluctuation range of 9°C (22), geosmin or 2-MIB production is expected to be high due to environmental conditions that are suitable for metabolite production. Therefore, the aim of the present study is to clarify potential odor-producing actinomycetes, effects of temperature, and responsible genes in potential candidates from tropical environment.

## Materials and Methods

### Sampling

Freshwater and sediment samples were collected from Titiwangsa Lake and Universiti Malaya Lake, which are located in Kuala Lumpur, Malaysia. Both are man-made lakes with an average depth of 10 meters and 6 meters for Titiwangsa Lake and Universiti Malaya Lake, respectively, and serve as recreational areas as well as habitats for aquatic life. Surface water samples were collected with a sampling container. Approximately 1 L of water was collected from each sampling location. Bank sediment samples were collected near the shoreline of the lakes using a scooping tool and the sampled sediment depth was approximately 30 cm. All samples were collected in duplicate and stored in dark glass bottles in an ice box before they were transferred to the laboratory. Water samples were held at 4°C, and sediment samples were stored at −20°C until used.

### Isolation of potential odor-producing actinomycetes

Since this study aimed to isolate potential odor-producing actinomycetes (mainly *Streptomyces* spp.), Basal Salts (BS) medium (KNO_3_, 2.0 g; K_2_HPO_4_, 0.5 g; MgSO_4_.7H_2_O, 0.4 g; FeSO_4_.7H_2_O, 0.1 g; starch, 10.0 g; agar, 20.0 g; amount L^−1^ pure water, pH 7.0) was selected for the growth of isolates because it is widely used to culture *Streptomyces* spp. (32, 33). Water and sediment samples were subjected to appropriate serial dilutions and then spread on BS agar plates. Inoculated plates were incubated at 30°C for 7 d until colonies were visible. Colonies with the typical actinomycetes morphology (dense and leathery appearance) were selected and screened by olfactory detection, color, and morphology under a microscope. Selected isolates were then purified on Yeast Meat Peptone Dextrose (YMPD) agar plates (yeast-extract, 2.0 g; meat-extract, 2.2 g; bacto-extract, 4.0 g; NaCl, 2.0 g; MgSO_4_.7H_2_O, 1.0 g; glucose, 1.0 g; agar, 20.0 g; amount L^−1^ pure water, pH 7.2) several times in order to obtain pure culture isolates. YMPD agar medium was used as a maintenance culture for the isolates and also medium for the production of geosmin and 2-MIB (11). Isolates were subjected to DNA extraction, sequencing, and temperature-controlled experiments in order to assess their geosmin- and 2-MIB-producing abilities.

### DNA extraction and polymerase chain reaction (PCR) amplification of the 16S rRNA gene and *geoA* and *tpc* genes

Purified single colonies grown on YMPD agar plates were collected, immersed in 100 μL of autoclaved water, and then mixed. An ultra-sonicator (Hielscher Ultrasound Technology, Teltow, Germany) was used to lyse cells using ultrasonic waves with a 0.5% cycle and 50% amplitude. DNA was then extracted from cell suspensions by adding 100 μL of phenol-chloroform-isoamyl alcohol (25:24:1) and gently mixing. After centrifugation at 20,600×*g* for 3 min, the upper layer of the suspension was collected, transferred to a new 1.5-mL tube, and stored at 4°C until analyzed. All primers used in the present study are shown in Table S1. Primers from Auffret *et al.* (2) and Giglio *et al.* (10) were used in the present study to detect the presence of *geoA* and *tpc* using PCR-based assays. The PCR amplification of the genes of interest, namely, the 16S rRNA gene (17), *geoA* (geosmin synthase gene) (10), and *tpc* (MIB synthase gene) (2), was performed in a volume of 20 μL with 5×Green GoTaq Flexi Buffer, 1.25 mM MgCl_2_, 0.2 mM dNTPs, 0.5 U GoTaq DNA polymerase, 0.5 μM of each primer (Promega, Madison, WI, USA), and 1 μL DNA template with the following protocol: 98°C for 2 min, followed by 35 cycles at 98°C for 30 s for *geoA* and *tpc* and 30 cycles at 95°C for 30 s for the 16S rRNA gene, at the specific annealing temperatures shown in Table S1 for 30 s, at 72°C for 1 min 30 s, and at 72°C for 5 min. PCR amplicons were verified by 0.8% agarose gel electrophoresis (100 V for 25 min), Diamond^TM^ Nucleic Acid Dye (Promega) staining, and visualization under an ultraviolet light using a Vilber Lourmat Quantum ST5 Gel Documentation System (Fisher Scientific, Waltham, MA, USA).

### Sequencing of the 16S rRNA gene

Purified 16S rRNA gene PCR products were sequenced with the universal primers 27F and 1492R using a BigDye Terminator v3.1 cycle sequencing kit (Applied Biosystems, Foster City, CA, USA) as recommended by the manufacturer. Purified mixtures were sequenced using an ABI PRISM 3730XL Genetic Analyzer. Using BLAST search software (http://blast.ncbi.nlm.nih.gov/Blast.cgi), known 16S rRNA gene sequences from actinomycetes were selected from the GenBank databases for comparisons with the sequences of isolates obtained in the present study. Sequences were compared with the sequences of representative actinomycetes in a multiple sequence alignment together with all gaps removed using the Clustal W program. The 16S rRNA gene nucleotide sequences identified in this study were deposited in the NCBI database with the following reference numbers: KY305476, KY305477, KY305478, KY305479, KY305480, and KY305481.

### Geosmin and 2-MIB production at different temperatures

Six purified isolates were tested for their abilities to produce geosmin and 2-MIB at different temperatures. Isolates were inoculated into 50 mL of YMPD liquid medium to prepare for the seed culture and incubated at 30°C for 5 d under shaking at 120 rpm. Cells were harvested by centrifugation (5,000×*g*, 25°C, 5 min) and were washed twice with dispersed BS negative medium ([NH_4_]_2_SO_4_, 0.2 g; NaCl, 0.2 g; K_2_HPO_4_, 0.05 g; MgSO_4_.7H_2_O, 0.1 g; FeSO_4_.7H_2_O, 0.005 g; amount 100 mL^−1^ pure water, pH 8.0) (11). One hundred and fifty microliters of the washed pellets was spread on YMPD agar plates. YMPD agar was used because it is favorable for supporting the development of spores. All plates were inverted and cultured at different temperatures for 7 d: 20°C, 25°C, 30°C, 35°C, and 40°C. After the 7-d incubation, 5 mL of methanol was added directly to each plate to cover the agar plate entirely and left at 25°C for 30 min for geosmin and 2-MIB extraction. One milliliter of the methanol geosmin/2-MIB extract was transferred to a glass tube with 2 mL of *n*-hexane added. The mixture was then incubated at 25°C for 30 min with shaking at 150 rpm, and then centrifuged (800×*g*, 25°C, 30 min) to separate the *n*-hexane and methanol layers. The upper *n*-hexane layer was carefully collected and dehydrated over anhydrous Na_2_SO_4_ in a Pasteur pipette (14). Extracts were held at −20°C prior to the gas chromatography-mass spectrometry analysis (GC-MS 7890A; Agilent Technologies, Santa Clara, CA, USA) to measure geosmin and 2-MIB concentrations.

### GC-MS analysis of volatile metabolites

Quantification was performed by integrating the base peak area. The original geosmin and 2-MIB standards (100 μg mL^−1^ in methanol) were purchased from Supelco (Sigma-Aldrich, Bellefonte, PA, USA) and diluted to 10^7^, 5×10^6^, 10^6^, 5×10^5^, and 10^5^ ng L^−1^ in order to generate calibration curves. A DB-WAX (30 m×0.25 μm×0.25 mm) column was used with He as the carrier gas at a constant flow of 1 mL min^−1^. An aliquot of 1 μL of each sample was injected into the inlet of each sample vial with a 5-min solvent delay. The inlet temperature was maintained at 250°C and operated in a split mode ratio of 5:1. The temperature program began at 50°C, was increased to 200°C with a ramp rate of 10°C min^−1^, then increased to 260°C at a rate of 30°C min^−1^ for 1 min. After using the full scan mode at a range of 50–300 *m/z*, geosmin and 2-MIB were quantified by their main characteristic ions, *m/z* 112 and *m/z* 95. Retention times were 11.29±0.3 min for geosmin and 8.19±0.4 min for 2-MIB. Geosmin and 2-MIB concentrations in the cultures were calculated by comparisons with the standard curve.

### Water quality analysis

The quality of water from Titiwangsa Lake was assessed and ambient temperature was measured. Ten parameters were assessed. The sampling site was at longitude 101°42′20.8152″E and latitude 3°10′35.6952″N. The *in situ* parameters, temperature, dissolved oxygen (DO), pH, turbidity, conductivity, and total dissolved solids (TDS), were measured using a multiparameter (YSI Pro Plus; YSI, Yellow Springs, OH, USA), whereas ammonia-N (NH_3_-N), nitrate (NO_3_^−^), nitrite (NO_2_^−^), and phosphate (PO_4_^3−^) were analyzed in the laboratory. The water sample was filtered through glass microfiber filters (45 mm in diameter, Life Sciences, UK) and 100 mL of filtrated water was placed into a 100-mL bottle for a nutrient analysis. The nutrient contents of samples were analyzed using a water quality testing kit according to the manufacturer’s instructions (Kyoritsu Chemical-Check Lab., Tokyo, Japan).

## Results

### Identification and characterization of geosmin/2-MIB-producing actinomycetes from the Tropics

Two sampling sites were selected because no musty odor was detected in Titiwangsa Lake and a musty odor was slightly recognized in Universiti Malaya Lake. Fifty candidates were initially obtained from the water and sediments. During the screening process, isolates with different odor intensities (strong, medium, and weak), different colored sporulating aerial mycelia (dark grey, greyish white, whitish, and greenish white), and the same characteristics of actinomycetes morphology common in the taxonomy of *Streptomyces* spp. (branching filamentous mycelia and rod-shape cocci) were selected. Six potential actinomycetes (Fig. S1) were selected from the screening process and genomic DNA from each strain was extracted. The 16S rRNA gene was amplified by PCR and analyzed by BLAST in the NCBI database (the 16S ribosomal DNA sequence database). Based on BLAST results (Table 1), six actinomycetes were affiliated with the genus *Streptomyces*, in which strain T-S1 showed 98% sequence similarity with *S. zaomyceticus*, strain T-S2 showed 95% sequence similarity with *S. hirsutus*, and strain T-S4 showed 97% sequence similarity with *S. sampsonii*. Strain T-S5 showed 96% sequence similarity, while strains U-S3 and U-S6 showed 97% sequence similarities with *S. coelicolor*. The ability of each isolate to produce geosmin and 2-MIB was detected by PCR-based assays using two molecular markers: *geoA*, the gene responsible for the production of geosmin, and *tpc*, the gene responsible for the production of 2-MIB. PCR products were at the expected sizes for the producers of geosmin (743 bp) and 2-MIB (592 bp), indicating that the primers used in this study specifically targeted *geoA* and *tpc*. All strains showed a clear band for the *geoA* gene (Fig. S2), whereas only strains T-S1 and T-S2 showed the band for the *tpc* gene, while the *tpc* gene was not amplified in strain U-S3, T-S4, T-S5, or U-S6 (Fig. S3).

### Effects of temperature on the production of geosmin and 2-MIB

**Fig. 1:**
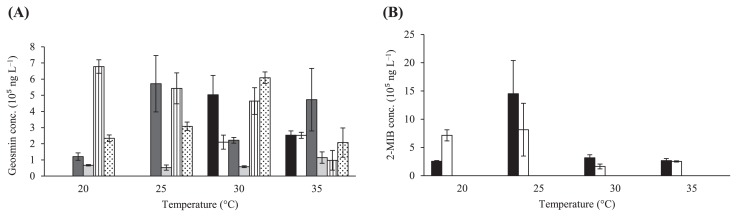
Effects of different temperatures (20°C, 25°C, 30°C, and 35°C) on mean geosmin concentrations (ng L^−1^) (A) and mean 2-MIB concentrations (ng L^−1^) (B) by all six strains. The black box represents strain T-S1; the open box represents strain T-S2; the horizontally lined box represents strain U-S3; the dashed lined box represents strain T-S4; the vertically lined box represents strain T-S5; and the circle-filled box represents strain U-S6. Error bars show the standard error, *n*=3.

Six isolated strains were cultured to investigate their odor-producing capacities depending on temperature. Among odor-producing actinomycetes, four strains (U-S3, T-S4, T-S5, and U-S6) exude geosmin only, whereas two strains (T-S1 and T-S2) exude geosmin and 2-MIB, which reflects genotypic distinction. Fig. 1A shows the effects of different temperatures on geosmin production by all six isolated strains. Only strains T-S1 and T-S2 showed no geosmin production at incubation temperatures of 20°C and 25°C. Strains U-S3, T-S4, T-S5, and U-S6 produced geosmin at all temperatures tested. At 20°C, strain T-S5 showed the highest production level of geosmin among all of the isolates with a yield of 6.8×10^5^ ng L^−1^ as well as the highest production at all temperatures tested. However, the amount of geosmin produced decreased as the temperature increased. Strain U-S3 produced the highest level of geosmin with a yield of 5.7×10^5^ ng L^−1^, followed by strains T-S5 and U-S6 at 25°C. Strain T-S4 showed the lowest amount of geosmin produced at 25°C. All isolates produced geosmin at 30°C, with the highest production level by strain U-S6. Strain T-S1 showed the next highest production level of geosmin, followed by strains T-S5, U-S3, T-S2, and T-S4. Geosmin production by strain U-S3 was higher than that by any other isolate after a 7-d incubation at 35°C. Strain T-S4 showed the lowest production level of geosmin among all isolates at all incubation temperatures. Fig. 1B shows the effects of different temperatures on 2-MIB production by all six isolated strains. Among the six strains tested, only strains T-S1 and T-S2 produced 2-MIB at all temperatures tested. The highest level of 2-MIB produced was observed by strain T-S1 at 25°C after a 7-d incubation, and higher levels of 2-MIB were produced among all incubation temperatures than geosmin. No marked differences were observed between 2-MIB production by strain T-S2 at 20°C and 25°C. Both isolates showed the least amount of 2-MIB produced at moderate temperatures of 30°C and 35°C.

Geosmin production showed a wide range of temperatures for each strain, but resulted in a narrow temperature range for 2-MIB production. Strains T-S1 and U-S6 resulted in optimal geosmin production at 30°C, whereas strains T-S2 and T-S4 showed optimal geosmin production at 35°C. Optimal geosmin production by strain U-S3 occurred at 25°C, while that by strain T-S5 occurred at 20°C. Regarding 2-MIB production, strains T-S1 and T-S2 showed optimal production at 25°C. Neither geosmin nor 2-MIB was detected after a 7-d incubation at 40°C by any of the isolated strains because no growth was observed on the plate at this incubation temperature.

### Water quality parameters

Water quality was measured over 4 months in order to prove the stability of temperature and obtain information on the quality of the lake. The parameters measured monthly in this study are shown in Table S2. Temperature was stable between September 2015 and January 2016, with a mean average temperature of 31.48°C and range between 30.48°C and 31.93°C. Overall, the results obtained showed acceptable values for the water quality parameters of Titiwangsa Lake, which were below the exceeded limits.

## Discussion

Numerous studies have been conducted on odorous compounds in freshwater or sources of drinking water in temperate and subtropical areas; however, few have investigated the production of geosmin and 2-MIB by actinomycetes in tropical areas. Since there have been no musty odor episodes reported at Titiwangsa Lake, it is interesting that the geosmin and 2-MIB producer strains T-S1, T-S2, T-S4, and T-S5 were successfully isolated from this sampling site. Musty odors have been slightly recognized at Universiti Malaya Lake, these producers were expected, and isolates U-S3 and U-S6 were obtained from this source. PCR assays also successfully amplified the *geoA* and *tpc* genes in our isolates, indicating that tropical actinomycetes possess geosmin and 2-MIB synthetic pathways as conserved pathways.

In the present study, all six isolated strains showed the ability to produce geosmin, whereas only two produced both geosmin and 2-MIB. Similar findings were reported by Zuo *et al.* (42), who measured geosmin and 2-MIB production from eight actinomycetes isolates from the sediments of the Xionghe Reservoir, China, grown in M liquid medium. In that study, six of the isolated actinomycetes produced geosmin only, one produced 2-MIB only (*S. lavendulae*), and only one produced both geosmin and 2-MIB (*Streptomyces* sp.). Odor production was also investigated in three types of liquid media (41). When actinomycetes were cultured in Gause, Tryptic Soy Broth (TSB), and M media, 3, 0, and 1 strains produced 2-MIB only; thirteen, eighteen, and twenty strains produced geosmin only; and 8, 5, and thirteen strains produced both geosmin and 2-MIB, respectively. Therefore, based on these findings, most isolates generate geosmin only, some produce both geosmin and 2-MIB, and a few produce 2-MIB only. However, in samples from a Canadian River, 47% of the isolated actinomycetes produced both geosmin and 2-MIB, 35% and 6% produced only geosmin or 2-MIB, respectively, whereas 12% did not produce any odorous compounds (40).

The effects of temperature on 2-MIB production by actinomycetes have been examined (30, 35). In both studies (30, 35), the highest production of 2-MIB occurred at incubation temperatures of 15°C and 30°C, respectively. In this study, strain T-S1 showed the highest production level of 2-MIB at 25°C, instead of 15°C and 30°C. The highest production level of geosmin occurred at 25°C from strain U-S3. There is no evidence in the literature of high levels of geosmin production at 25°C. All isolates produced geosmin at 30°C and 35°C, with the highest levels of geosmin being produced by strain U-S6 at 30°C and by strain U-S3 at 35°C. This is a unique feature of isolates in the present study because all isolates showed geosmin production at high temperatures, revealing the strong tropical characteristics of these isolates. A previous study (3) on the comparative physiology of geosmin production by *S. halstedii* revealed that maximum geosmin production occurred at 35°C. The present study verified this finding: the highest production levels of geosmin occurred at 35°C from strain U-S3. In the study by Blevins *et al.* (3), no geosmin production or biomass was detected at 40°C, which is similar with the results of this study, in which no isolates produced any geosmin or 2-MIB at 40°C. Strains T-S1, U-S3, and U-S6 may be regarded as producers of 2-MIB and geosmin at warmer temperatures because they may produce more geosmin/2-MIB in Malaysia because of its tropical climate.

Based on the results of this study, a range of optimal production temperatures was found for each strain. Strain T-S5, for example, showed very high geosmin production levels at 20°C. Since Malaysia has a tropical climate with a stable temperature of approximately 27°C throughout the year, and temperature rarely drops below 20°C, this is an interesting result. This strain may have a unique characteristic that allows it to produce large amounts of geosmin at low temperatures. Furthermore, the ability to survive at a low temperature (20°C) observed in all strains is a unique and novel result that contradicts previous findings of larger production at higher temperatures or in warmer seasons. Some of the isolated strains (strains T-S1, U-S3, and U-S6) in the present study also survived at high temperatures (25°C, 30°C, and 35°C) and showed the highest production in this temperature range. Therefore, temperature may not be the only key factor influencing the production of geosmin and 2-MIB because different optimal production temperatures were observed among the strains. Strains T-S1 and T-S5 showed unique abilities to sense temperature stress because the optimal temperatures for their production of geosmin differed from those of isolates obtained in other studies. Regarding strain T-S5, it produced the largest amount of geosmin at a low temperature that does not reflect the climate in Malaysia. Therefore, a draft genome analysis of these isolates will be conducted in the future for comparisons with the known genes or enzymes of the complete pathway in order to identify missing or added genomic contents of both isolates.

Table 2 shows the comparison results for the detection of *geoA* and *tpc* with metabolite detection at different temperatures by a GC-MS analysis. The results of the GC-MS analysis were consistent with the PCR amplification of geosmin synthase and MIB synthase. The PCR amplification of several genes responsible for the synthesis of geosmin and 2-MIB has been widely used to detect the presence of geosmin- and 2-MIB-producing microbes in environmental and culture samples (2, 13, 15, 19, 36). If the gene amplifies, then it may be concluded that producers exist within the water bodies, thereby leading to the early detection of potential odor-producing actinomycetes. Strains T-S1 and T-S2 showed the presence of *geoA* and *tpc*. However, geosmin production was not observed for either isolate at 20°C or 25°C, even though *geoA* was detected. This result indicates that *geoA* is not expressed at these temperatures to produce geosmin (possibly due to a gene mutation). According to Kutovaya and Watson (15), this may be due to the production of a geosmin synthase protein or direct photosynthate into terpenoid metabolism from strains possessing *geoA*. Gene expression analyses will be focused on to elucidate the regulatory mechanisms of off-flavor production in future studies that assess not only the effects of temperature, but also other environmental factors on the biochemical regulation of geosmin and 2-MIB.

Musty odor production may reduce the quality of water, particularly in areas in which surface water is used as a source of drinking water (31). Based on the number of factors influencing the growth of *Streptomyces* spp. and geosmin/2-MIB production, the identification of local environmental conditions will provide valuable information. The data presented in Table S2 showed that temperature remained stable across the study months, with an average temperature of 31.48°C, which is considered to be suitable for the growth of *Streptomyces* spp. However, from the results obtained, temperature does not appear to be a key parameter for the production of geosmin in the Tropics, even with water showing a high temperature of 31.48°C. Since temperature effects may be avoided based on the results of the present study, we strongly recommend other environmental parameters, such as nutrient contents, as the focus of future studies to assess the production of geosmin/2-MIB in tropical regions with sustained high temperatures.

The results obtained in this study showed that isolates exhibited various optimum temperatures for the production of these compounds. Our results also demonstrated that known geosmin and 2-MIB synthetic pathway genes have already spread into the environment in Malaysia. Based on water quality results, water temperature in the natural environment was approximately 31°C, and together with the higher production of geosmin from some isolates at 30°C, there is a strong possibility of musty odor episodes here in the future due to the moderate and humid climate conditions, which are suitable for the production of geosmin/2-MIB and growth of microbes throughout the year. To the best of our knowledge, the effects of environmental factors on the production of musty odors in water environments in Southeast Asia have not yet been investigated, even though moderate temperatures (approximately 30°C) in seasonal areas are associated with the production of these metabolites. Previous studies reported high geosmin production levels at temperatures ranging between 25°C and 35°C, which may explain the higher concentrations of the compound observed during warmer months (4, 35, 37, 38). However, the results of this study disagree with this expectation. There is still a paucity of information on taste and odor outbreaks by actinomycetes in tropical areas, particularly in Southeast Asia; however, we successfully isolated potential candidates for actinomycetes producers that also possessed the responsible genes, *geoA* and *tpc*. A clearer understanding of the impact of environmental conditions on geosmin and 2-MIB production together with molecular approaches may provide further insights into the contribution of actinomycetes to off-flavor occurrences in water bodies in the Tropics.

## Supplementary information



## Figures and Tables

**Table 1:** Results of a BLAST analysis of 16S rRNA gene sequences from six actinomycetes strains.

Strain	Sequence (bp)	Closest hit in GenBank (Accession no.)	Strain no.	Similarity (%)
T-S1	1271	*Streptomyces zaomyceticus* NR 044144.1	NRRL B-2038	98
T-S2	1121	*Streptomyces hirsutus* NR 043819.1	NRRL B-2713	95
U-S3	1309	*Streptomyces coelicolor* NR 116633.1	DSM 40233	97
T-S4	1162	*Streptomyces sampsonii* NR 116508.1	NRRL B12325	97
T-S5	1305	*Streptomyces coelicolor* NR 116633.1	DSM 40233	96
U-S6	1331	*Streptomyces coelicolor* NR 116633.1	DSM 40233	97

**Table 2:** Characterization of actinomycetes strains. Detection of *geoA* with the primer pair 250F/971R and *tpc* with the primer pair AMmib-F/AMmib-R by PCR and odorous metabolites by gas chromatography at different temperatures.

Strain	Closest hit in GenBank	PCR		20°C		25°C		30°C		35°C	
				
		*geo A*	*tpc*	G	M	G	M	G	M	G	M
T-S1	*Streptomyces zaomyceticus*	+	+	−	+	−	+	+	+	+	+
T-S2	*Streptomyces hirsutus*	+	+	−	+	−	+	+	+	+	+
U-S3	*Streptomyces coelicolor*	+	−	+	−	+	−	+	−	+	−
T-S4	*Streptomyces sampsonii*	+	−	+	−	+	−	+	−	+	−
T-S5	*Streptomyces coelicolor*	+	−	+	−	+	−	+	−	+	−
U-S6	*Streptomyces coelicolor*	+	−	+	−	+	−	+	−	+	−

+; Detected in PCR or GC-MS, −; Not detected in PCR or GC-MS

G=geosmin, M=2-MIB

## References

[1] Asquith E.A., Evans C.A., Geary P.M., Dunstan R.H., Cole B. (2013). The role of actinobacteria in taste and odour episodes involving geosmin and 2-methylisoborneol in aquatic environments. J Water Supply: Res Technol —AQUA.

[2] Auffret M., Pilote A., Proulx E., Proulx D., Vanderberg G., Villemur R. (2011). Establishment of real time PCR method for quantification of geosmin-producing *Streptomyces* spp. in recirculating aquaculture systems. Water Res.

[3] Blevins W.T., Schrader K.K., Saadoun I. (1995). Comparative physiology of geosmin production by *Streptomyces halstedii* and *Anabaena* sp. Water Sci Technol.

[4] Bruchet A. (1999). Solved and unsolved cases of taste and odor episodes in the files of inspector Cluzeau. Water Sci Technol.

[5] Che Rohani A., Normah O., Zahrah T., Che Utama C.M., Saadiah I. (2009). Quality of fish fillet from pond-raised red tilapia and its utilization in the development of value-added product. J Trop Agric Food Sci.

[6] Dickschat J.S., Nawrath T., Thiel V., Kunza B., Müller R., Schulz S. (2007). Biosynthesis of the off-flavor 2-methylisoborneol by the Myxobacterium *Nannocystis exedens*. Angewandte Chem.

[7] Dionigi C., Ingram D. (1994). Effects of temperature and oxygen concentration on geosmin production by *Streptomyces tendae* and *Penicillium expansum*. J Agric Food Chem.

[8] Gerber N.N., Lechevalier H.A. (1965). Geosmin, an earthly-smelling substance isolated from actinomycetes. Appl Microbiol.

[9] Gerber N.N. (1969). A volatile metabolite of actinomycetes, 2-methylisoborneol. J Antibiot.

[10] Giglio S., Jiang J., Saint C.P., Cane D.E., Monis P.T. (2008). Isolation and characterization of the gene associated with geosmin production in cyanobacteria. Environ Sci Technol.

[11] Hikida T., Shimizu K., Umeta T., Utsumi M., Sugiura N. (2012). Effect of starvation on musty odor production by *Streptomyces coelicolor* A3 (2). J Bioindust Sci.

[12] Izaguirre G., Hwang C.J., Krasner S.W., Mcguire M.J. (1982). Geosmin and 2-metyhlisoborneol from cyanobacteria in three water supply systems. Appl Environ Microbiol.

[13] Jorgensen N.O.G., Podduturi R., Burford M.A. (2016). Relations between abundance of potential geosmin- and 2-MIB-producing organisms and concentrations of these compounds in water from three Australian reservoirs. J Water Supply: Res Technol—AQUA.

[14] Komatsu M., Tsuda M., Omura S., Oikawa H., Ikeda H. (2008). Identification and functional analysis of genes controlling biosynthesis of 2-methylisoborneol. Proc Natl Acad Sci USA.

[15] Kutovaya O.A., Watson A.B. (2014). Development and application of a molecular assay to detect and monitor geosmin-producing cyanobacteria and actinomycetes in the Great Lakes. J Great Lakes Res.

[16] La Guerche S., Garcia C., Darriet P., Dubourdieu D., Labarère J. (2004). Characterization of *Penicillium* species isolated from grape berries by their Internal Transcribed Spacer (ITS1) sequences and by Gas Chromatography-Mass Spectrometry analysis of geosmin production. Curr Microbiol.

[17] Lane D.J., Stackebrandt M., Goodfellow M. (1991). 16S/23S rRNA sequencing. Nucleic Acid Techniques in Bacterial Systematics.

[18] Lu G., Edwards C.G., Fellman J.K., Mattinson D.S., Navazio J. (2003). Biosynthetic origin of geosmin in red beets (*Beta vulgaris L.*). J Agric Food Chem.

[19] Ludwig F., Medger A., Börnick H., Opitz M., Lang K., Göttfret M., Röske I. (2007). Identification and expression analysis of putative sesquiterpene synthase genes in *Phormidium* sp. and prevalence of geoA-like genes in a drinking water reservoir. Appl Environ Microbiol.

[20] Mattheis J.P., Roberts R.G. (1992). Identification of geosmin as a volatile metabolite of *Penicillium expansum*. Appl Environ Microbiol.

[21] Medsker L., Jenkins D., Thomas J. (1968). Odorous Compounds in Natural Waters. An earthy-smelling compound associated with blue-green algae and actinomycetes. Environ Sci Technol.

[22] Nazeri M., Jusoff K., Madani N., Mahmud A.R., Bahman A.R., Kumar L. (2012). Predictive modeling and mapping of Malayan Sun Bear (*Helarctos malayanus*) distribution using maximum entropy. PloS One.

[23] Negoro T., Ando M., Ichikawa N. (1988). Blue-green algae in lake biwa which produce earthy-musty odors. Water Sci Technol.

[24] Nurul Izzah A., Ahmad F.B.H., Jamilah B., Salmah Y. (2004). Geosmin and isoborneol in Black Tilapia (*Orechromis mossambica*). Ultra Sci Phys Sci.

[25] Rashash D.M., Dietrich A.M., Hoehn R.C., Parker B.C. (1995). The influence of growth conditions on odor-compound production by two chrysophytes and two cyanobacteria. Water Sci Technol.

[26] Saadoun I. (2005). Production of 2-Methylisoborneol by *Streptomyces violaceusniger* and its transformation by selected species of *Pseudomonas*. J Basic Microbiol.

[27] Safferman R.S., Rosen A.A., Mashni C.I., Morris M.E. (1967). Earthy-smelling substance from a blue-green alga. Environ Sci Technol.

[28] Schrader K.K., Blevins W.T. (1993). Geosmin producing species of *Streptomyces* and *Lyngbya* from aquaculture ponds. Can J Microbiol.

[29] Schrader K.K., Blevins W.T. (2001). Effects of carbon source, phosphorus concentration, and several micronutrients on biomass and geosmin production by *Streptomyces halstedii*. J Ind Microbiol Biotechnol.

[30] Schrader K.K., Harries M.D., Page P.N. (2015). Temperature effects on biomass, geosmin, and 2-methylisoborneol production and cellular activity by *Nocardia* spp. and *Streptomyces* spp. isolated from rainbow trout recirculating aquaculture systems. J Ind Microbiol Biotechnol.

[31] Smith V.H., Sieber-Denlinger J., de Noyelles F., Campbell S., Pan S., Randtke S.J., Blain G.T., Strasser V.A. (2002). Managing taste and odor problems in a eutrophic drinking water reservoir. Lake Reservoir Manage.

[32] Sugiura N., Yagi O., Sudo R. (1983). Effect of various environmental factors in musty odor production by *Streptomyces*. Jpn J Water Pollut Res.

[33] Sugiura N., Nakano K. (2000). Causative microorganisms for musty odor occurrence in the eutrophic Lake Kasumigaura. Hydrobiologia.

[34] Trowitzsch W., Witte L., Reichenbach H. (1981). Geosmin from earthy smelling cultures of *Nannocystis exedens* (Myxobacterales). FEMS Microbiol Lett.

[35] Tung S.C., Lin T.F., Yang F.C., Liu C.L. (2008). Seasonal change and correlation with environmental parameters for 2-MIB in Feng-Shen Reservoir, Taiwan. Environ Monit Assess.

[36] Wang Z., Xu Y., Shao J., Wang J., Li R. (2011). Genes associated with 2-methylisoborneol biosynthesis in cyanobacteria: isolation, characterization, and expression in response to light. PLoS ONE.

[37] Westerhoff P., Rodriguez-Hernandez M., Baker L., Sommerfeld M. (2005). Seasonal occurrence and degradation of 2-methylisoborneol in water supply reservoirs. Water Res.

[38] Yagi M. (2005). 35 years history of off-flavor problems in the southern basin of Lake Biwa.

[39] Young W., Horth H., Crane R., Ogden T., Arnott M. (1996). Taste and odor threshold concentrations of potential potable water contaminants. Water Res.

[40] Zaitlin B., Watson S.B., Dixon J., Steel D. (2003). Actinomycetes in the elbow river basin, Alberta, Canada. Water Qual Res J Can.

[41] Zuo Y., Li L., Wu Z., Sohng L. (2009). Isolation, identification and odour-producing abilities of geosmin/2-MIB in actinomycetes from sediments in Lake Lotus, China. J Water Supply: Res Technol – AQUA.

[42] Zuo Y., Li L., Zhang T., Zheng L., Dai G., Liu L., Song L. (2010). Contribution of streptomyces in sediment to earthy odor in the overlying water in Xionghe Reservoir, China. Water Res.

