# Supplementary material for: Characterization of Musty Odor-Producing Actinomycetes from Tropics and Effects of Temperature on the Production of Musty Odor Compounds
Source: Microbes Environ. 2017 Oct 31;32(4):352–7. doi: 10.1264/jsme2.ME17109 (PMC5745020; doi:10.1264/jsme2.ME17109)
Supplement: Supplementary file 1 [file 32_352_s1.pdf]

Supplementary information

Table S1. Primer pairs used for PCR amplification.

Primer	Target gene	Sequences (5` - 3`)	Product length (bp)	Annealing temp. (°C)	Reference
27F<br>1492R	16S rRNA	AGAGTTTGATCCTGGCTCAG<br>GGTTACCTTGTTACGACTT	1,465	55	(1)
AMmib-F<br>AMmib-R	*tpc*	TGGACGACTGCTACTGCGAG<br>AAGGCGTGCTGTAGTTCGTTGTG	592	58	(2)
250F<br>971R	*geoA*	TTCTTCGACGAYCACTTCC<br>CCCTYGTTCATGTARCGGC	743	55	(3)

Table S2: Summary of water quality parameters for over four months of sampling from Titiwangsa Lake.

**Parameters**	**September (2015)**	**October (2015)**	**November (2015)**	**January (2016)**
Temperature (°C) (mean)	31.93	30.48	31.66	31.86
pH	8.11	8.69	7.7	8.48
DO (mg/L)	6.06	7.35	8.1	6.68
Turbidity (NTU)	5.1	4.9	4.9	103.0
Conductivity (µS/cm)	157.0	158.0	149.0	236.0
TDS (g/L)	0.099	0.103	0.097	0.151
Chl-a (µg/L)	4.634	5.566	3.079	2.515
Nitrate (mg/L)	ND	0.128	0.085	ND
Nitrite (mg/L)	0.003	0.001	0.007	0.002
Ammonia (mg/L)	ND	ND	ND	ND
Phosphate (mg/L)	0.003	0.003	0.028	ND

ND = not detected

Supplemental figure legends

Fig.S1 Six purified actinomycetes strains cultivated on YMPD agar plate. a) T-S1, b) T-S2, c) U-S3, d) T-S4, e) T-S5, and f) U-S6.

Fig.S2 Results of electrophoresis of amplified *geoA* from six isolated strains by PCR primed with 250F and 971R at an annealing temperature of 55°C. a) Lane 1 = T-S1; lane 4 = T-S2; lane 7 = U-S3 b) lane 1 = T-S4; lane 4 = T-S5; lane 7 = U-S6. Lanes 2, 5, and 8 and lanes 3, 6, and 9 show results of the isolates diluted four- and eight-fold, respectively.

Fig. S3 Results of electrophoresis of the target *tpc* amplified from six isolated strains by PCR primed with AMmib-F and AMmib-R at an annealing temperature of 58°C. (a) Lane 1 = T-S1; lane 4 = T-S2; lane 7 = U-S3 (b) lane 1 = T-S4; lane 4 = T-S5; lane 7 = U-S6. Lanes 2, 5, and 8 and lanes 3, 6, and 9 show results of the isolates diluted four- and eight-fold, respectively.

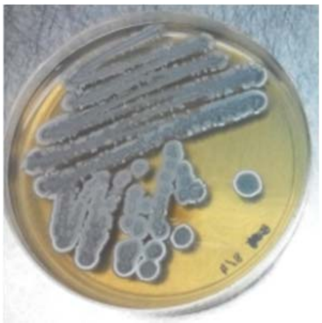

(a)

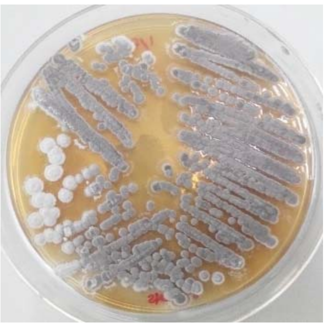

(b)

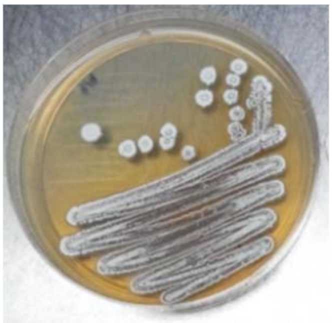

(c)

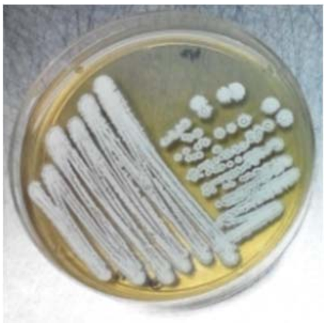

(d)

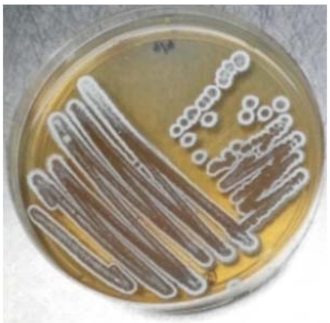

(e)

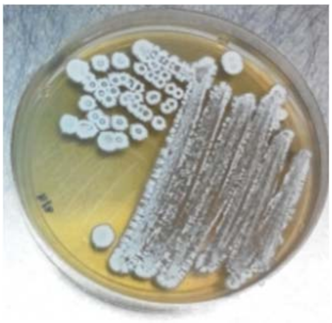

(f)

Fig. S1 Nurul Syahirah Shamsol Anuar et al.,

(a)

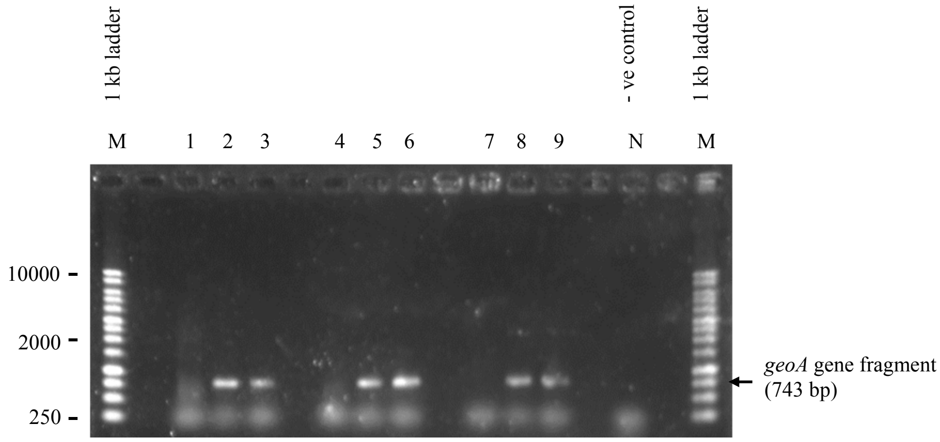

(b)

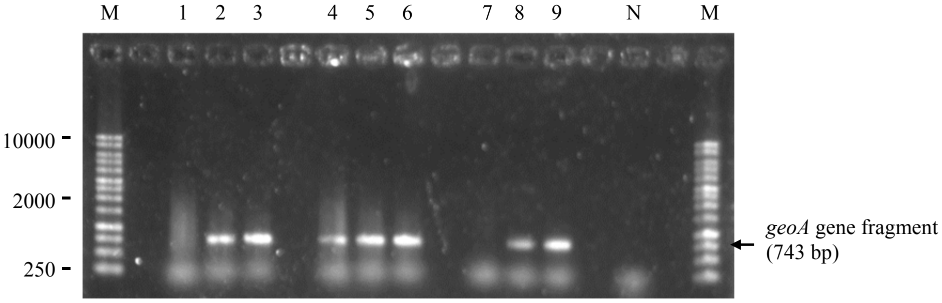

Fig. S2 Nurul Syahirah Shamsol Anuar et al.,

(a)

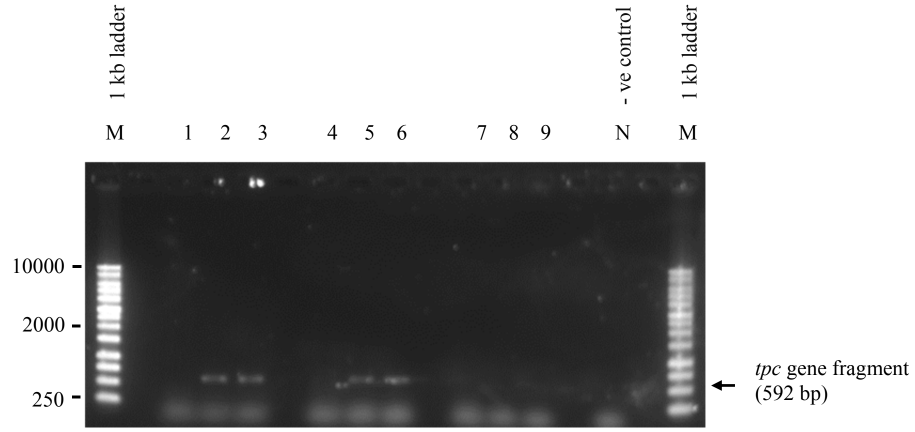

(b)

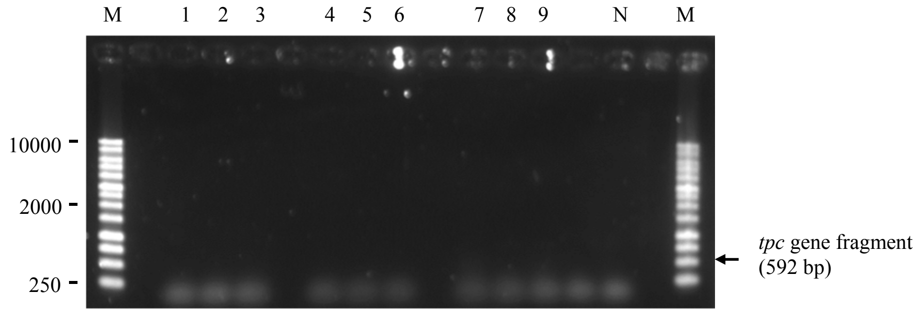

Fig. S3 Nurul Syahirah Shamsol Anuar et al.,

References for supplemental data

1. Lane, D. J. 1991. 16S/23S rRNA sequencing, p. 115–175. *In* M. Stackebrandt and M. Goodfellow (ed.), Nucleic Acid Techniques in Bacterial Systematics.Wiley, New York.
2. Auffret, M., A. Pilote, E. Proulx, D. Proulx, G.Vanderberg, and R. Villemur. 2011. Establishment of real time PCR method for quantification of geosmin-producing *Streptomyces* spp. in recirculating aquaculture systems. Water Research. 45:6762–6753.
3. Giglio, S., J. Jiang, C. P. Saint, D. E. Cane, and P.T. Monis. 2008. Isolation and characterization of the gene associated with geosmin production in cyanobacteria. Environmental Science & Technology. 42:8027–8032.
